# COVID-19 rapid molecular point-of-care testing is effective and cost-beneficial for the acute care of trauma patients

**DOI:** 10.1007/s00068-022-02091-x

**Published:** 2022-09-06

**Authors:** Josef Stolberg-Stolberg, Elena Jacob, Joachim Kuehn, Marc Hennies, Wali Hafezi, Moritz Freistuehler, Jeanette Koeppe, Alex W. Friedrich, J. Christoph Katthagen, Michael J. Raschke

**Affiliations:** 1 Department of Trauma, Hand and Reconstructive Surgery, University Hospital Muenster, Albert-Schweitzer-Campus 1, Building W1, 48149 Muenster, Germany; 2 Department of Clinical Virology, Institute of Virology, University Hospital Muenster, 48149 Muenster, Germany; 3 Medical Management Division-Medical Controlling, University Hospital Muenster, Niels-Stensen-Straße 8, 48149 Muenster, Germany; 4 Institute of Biostatistics and Clinical Research, University of Muenster, Schmeddingstrasse 56, 48149 Muenster, Germany; 5 Medical Executive Board, University Hospital Muenster, Albert-Schweitzer-Campus 1, Building D5, 48149 Muenster, Germany

**Keywords:** COVID-19, Rapid molecular point-of care testing, ID NOW, Traumatology, Emergency medicine

## Abstract

**Purpose:**

To evaluate the accuracy and cost benefit of a rapid molecular point-of-care testing (POCT) device detecting COVID-19 within a traumatological emergency department.

**Background:**

Despite continuous withdrawal of COVID-19 restrictions, hospitals will remain particularly vulnerable to local outbreaks which is reflected by a higher institution-specific basic reproduction rate. Patients admitted to the emergency department with unknown COVID-19 infection status due to a- or oligosymptomatic COVID-19 infection put other patients and health care workers at risk, while fast diagnosis and treatment is necessary. Delayed testing results in additional costs to the health care system.

**Methods:**

From the 8th of April 2021 until 31st of December 2021, all patients admitted to the emergency department were tested with routine RT-PCR and rapid molecular POCT device (Abbott ID NOW™ COVID-19). COVID-19-related additional costs for patients admitted via shock room or emergency department were calculated based on internal cost allocations.

**Results:**

1133 rapid molecular tests resulted in a sensitivity of 83.3% (95% CI 35.9–99.6%), specificity of 99.8% (95% CI 99.4–100%), a positive predictive value of 71.4% (95% CI 29–96.3%) and a negative predictive value of 99.9% (95% CI 99.5–100%) as compared to RT-PCR. Without rapid COVID-19 testing, each emergency department and shock room admission with subsequent surgery showed additional direct costs of 2631.25€, without surgery of 729.01€.

**Conclusion:**

Although rapid molecular COVID-19 testing can initially be more expensive than RT-PCR, subsequent cost savings, improved workflows and workforce protection outweigh this effect by far. The data of this study support the use of a rapid molecular POCT device in a traumatological emergency department.

## Introduction

As by March 2022, approximately 450 million cases of severe acute respiratory syndrome coronavirus 2 (SARS-CoV-2) infection, 6 million deaths associated with the corona virus disease 2019 (COVID-19) and 10 billion administered vaccine doses have been reported to the World Health Organization [1]. Despite collective efforts, the number of new cases increased markedly during the first week of the new year with 78% and 31% in the region of the Americas and Europe, respectively [2]. Hence, organizational actions to guarantee functioning orthopaedic trauma services are needed. The aim of this study is to evaluate the clinical use of a rapid molecular point-of-care testing (POCT) device as compared to conventional reverse transcription polymerase chain reaction (RT-PCR) testing for SARS-CoV-2 detection.

The Abbott ID NOW™ COVID-19 system is a POCT device using isothermal nucleic acid amplification technology for qualitative detection of SARS-CoV-2 RNA. It amplifies the RNA-dependent RNA polymerase (RdRp) viral target gene with a claimed limit of detection (LOD) of 125 genome equivalents/ml [3]. Using an upper respiratory tract swab, positive results can be available as soon as 5 min and negative results within 13 min [4]. Although the LOD of RT-PCR is lower than that of ID NOW™, recent systematic reviews suggest that ID NOW™ is effective in identifying or excluding SARS-CoV-2 in symptomatic ambulatory populations [5]. While cost–benefit analyses of other POCT devices such as for influenza A and B have been shown to be effective and economic for clinical decision-making in an emergency department, so far, there are limited data concerning SARS-CoV-2 [6, 7]. SARS-CoV-2 antigen testing already showed major cost savings particularly due to reduced unnecessary bed blocking [8]. Hence, the aim of this study is to assess testing effectiveness of a molecular SARS-CoV-2 POCT device and to model cost-beneficial effects within an acute care trauma center.

## Patients and methods

To verify the accuracy of Abbot ID NOW™ COVID-19 detection, we performed routine SARS-CoV-2 RT-PCR in addition to ID NOW™ testing from the 8th of April 2021, the day the ID NOW™ device was first implemented at the level 1 trauma center of the University Hospital Muenster, until the 31st of December 2021. During this period, 1133 ID NOW™ tests were conducted on trauma patients that met one of the following criteria: admission via shock room or admission via the regular emergency department (ED) due to the necessity of in-patient treatment or urgent surgery. All test samples were collected by trained health care professionals. Nasal swabs were collected from both nostrils, placed into swab transport solution (Sigma Transwab liquid Amies), and kept at room temperature. ID NOW™ testing was performed immediately. RT-PCR samples were transported to the virology laboratory with a maximum delay of 12 h and tested with Altona diagnostics RealStar SARS-CoV-2 RT-PCR according to the manufacturer’s instructions.

We compared the additional effort and expenses during daily procedures in the trauma department that had been necessary due to the pandemic and evaluated, how much it could be reduced due to the introduction of ID NOW™ POCT. Both scenarios, with and without ID NOW™ testing, were broken down into diagnostic and treatment steps. Material costs of COVID-19 personal protective equipment (PPE), e.g., masks, face shields, gowns, gloves, and the costs of the available COVID-19 testing strategies, according to internal cost allocation were provided by our in-house finance-controlling department. Due to the complexity of hospital billing, certain costs and efforts such as time saved, elevated cleaning costs and increased stress on employees could only be described and not expressed in total numbers.

Statistical analyses were performed using SAS software V9.4, SAS Institute Inc., Cary, NC, USA. All analyses were fully explorative and all results are interpreted accordingly. 95% Confidence intervals (95% CIs) were given by exact binominal limits.

## Results

During the examined time, 1133 ID NOW™ COVID-19 tests were performed. Seven ID NOW™ tests had a positive result, of which five were verified by RT-PCR. Therefore, 2 ID NOW™ results were false-positive. 1126 ID NOW™ tests had a negative result, which was correct according to the RT-PCR test in all but one cases (Table 1). This patient with false-negative result initially showed a *C*_T_-value of 37.43 which decreased to 20.9 2 days later indicating a very early stage of infection. Overall, the analysis of 1133 patients resulted in a sensitivity of 83.33% (95% CI 35.88–99.58%), specificity of 99.82% (95% CI 99.36–99.98%), a positive predictive value of 71.43% (95% CI 29.04–96.33%), and a negative predictive value of 99.91% (95% CI 99.51–100%).

**Table 1:** Performance of ID NOWTM in diagnosis of COVID-19

	Disease present	Disease absent	Total
ID NOW™ positive	5	2	7
ID NOW™ negative	1	1125	1126
Total	6	1127	1133

To account for possible cost savings due to the introduction of POCT device ID NOW™, additional costs for the admission via the ED or shock room were calculated as follows: internal cost allocations were provided by the controlling department of the hospital and are based on bulk orders and price levels during the study period: RT-PCR 41.10€, ID NOW™ 50.25€, PPE 4.54€ (gown 1.85€, pair of gloves 0.18€, hair net 0.06€, FFP2 face mask 0.55€, face shield 1.9€), lump sum empty containment bed in a two bedroom 710€/day, and average costs operating room (OR) per min 25€/min (Table 2). An average of 75 min post-anaesthetic care unit length of stay was calculated as basis for additional OR time, if the patient needed to remain in the OR due to undefined COID-19 status [9].

**Table 2:** Direct additional costs of COVID-19 testing, PPE, and isolation

	Costs ID NOW™		Costs RT-PCR
Initial patient screening:			
ED	Nurse, physician PPE 9.08€		Nurse, physician PPE 9.08€
Shock room	Trauma team PPE 40.86€		Trauma team PPE 40.86€
Testing	50.25€		41.10€
Imaging	0 €		Nurse PPE 4.54€ Radiology technician PPE 4.54€
Patient consultation and preparation for surgery/ward	0 €		Nurse PPE 4.54€ Physician PPE 4.54€
Surgery	0 €		2 physicians PPE 9.08€ 2 surgery nurses PPE 9.08€ Anaesthesiologist PPE 4.54€ Anaesthesia nurse PEE 4.54€
Post-anaesthesia care in the OR	0 €		1875€
Empty containment bed	0 €		720€
Total			
ER admission			
With surgery	59.33€		2690.58€
Without surgery	59.33€		788.34€
SR admission			
With surgery	91.11€		2722.36€
Without surgery	91.11€		820.12€

**Fig. 1:**
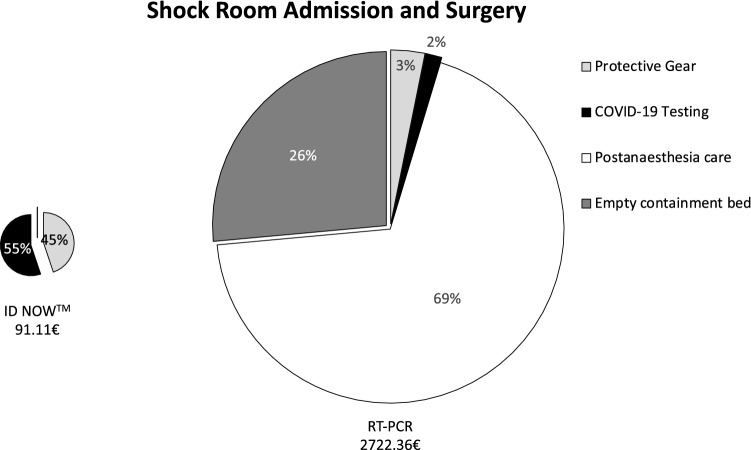
COVID-19-related additional costs of 2722.36€ for RT-PCR and 91.11€ for rapid molecular testing are calculated in the case of a shock room admission and subsequent surgery. Rapid molecular testing results in cost savings of 2631.25€

**Fig. 2:**
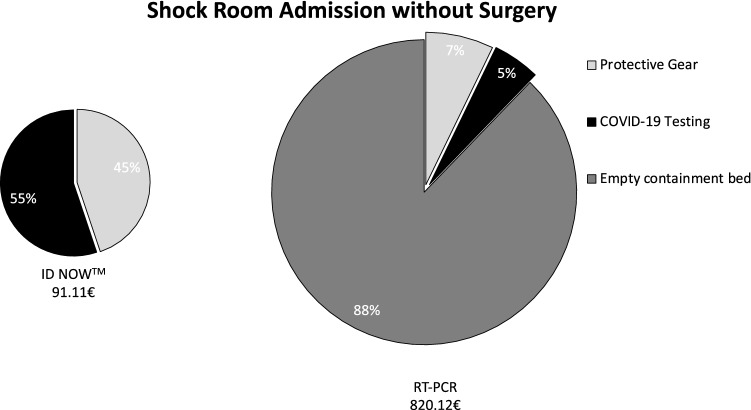
COVID-19-related additional costs of 820.12€ for RT-PCR and 91.11€ for rapid molecular testing are calculated in the case of a shock room admission without surgery. Rapid molecular testing results in cost savings of 729.01€

**Fig. 3:**
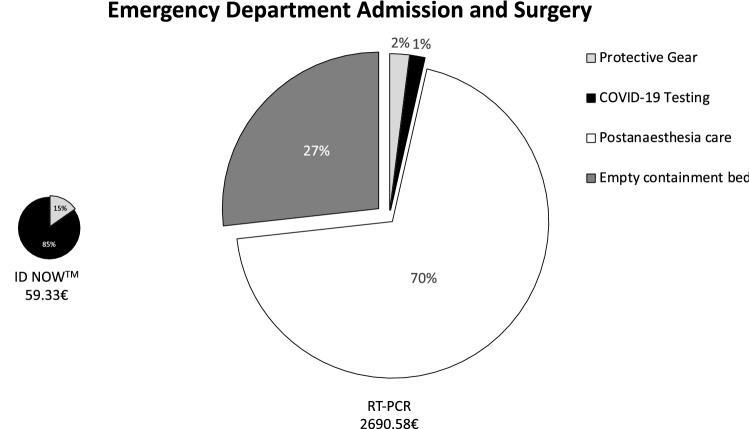
COVID-19-related additional costs of 2690.58€ for RT-PCR and 59.33€ for rapid molecular testing are calculated in the case of a regular emergency department admission and subsequent surgery. Rapid molecular testing results in cost savings of 2631.25€

**Fig. 4:**
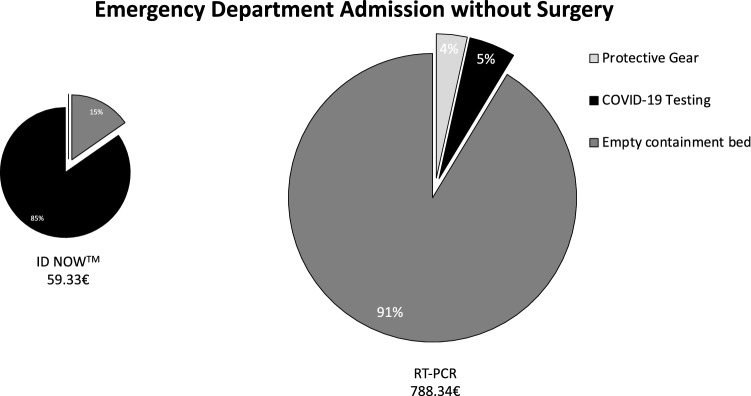
COVID-19-related additional costs of 788.34€ for RT-PCR and 59.33€ for rapid molecular testing are calculated in the case of a regular emergency department admission without surgery. Rapid molecular testing results in cost savings of 729.01€

Briefly, when a patient is brought to the shock room, an interdisciplinary team, consisting of at least nine people (trauma surgeon, abdominal surgeon, anaesthesiologist, anaesthesia nurse, neurosurgeon, radiologist, radiology technician, and two nurses), must wear PPE, resulting in a total of 40.86€. Without POCT, radiological technicians (9.08€) must again wear PPE for CT scan before a nurse and physician can prepare the patient for surgery or ward, so that again protection (9.08€) is necessary. Within the OR, two surgeons, two surgery nurses, and anaesthesia team need protection during surgery (27.24€). After surgery, the post-anaesthetic care must be provided within the OR as the patient cannot be brought to the recovery room, resulting in an average cost of 25€/min, multiplied by an average length of stay of 75 min which amounts to total additional costs of 1875€. Until the RT-PCR test results show negative, the bed next to the patient must be kept empty adding another 720€. In total, using only RT-PCR a shock room admission with subsequent surgery results in additional direct costs of 2631.25€ (Fig. 1), without surgery of 729.01€ (Fig. 2). In the case of a regular admission via the emergency department, the initial team of nurse and physician needs PPE (9.08€), which is not anymore required once rapid molecular testing is conducted. In the case of surgery, the above described additional costs can be avoided, resulting in total costs of 59.33€ in the case of rapid molecular testing and 2690.58€ for RT-PCR. Without surgery, 788.34€ are calculated for patients without as opposed to 59.33€ with POCT. Consequently, a total of 2631.25€ can be saved for patients needing surgery (Fig. 3) and 729.01€ for patients not requiring surgical intervention (Fig. 4).

## Discussion

The main findings of this study are that major cost savings and process improvements of trauma patients delivered to both the shock room and ED can be achieved by the POCT. Furthermore, test results confirm high sensitivity and specificity described in literature.

Numerous studies investigated the COVID-19 diagnosis agreement between ID NOW™ and RT-PCR [10–13]. A recent review published by Tu et al*.* including 15 studies with at least 20 subjects summarized an overall ID NOW™ sensitivity of 84% (95% CI 55–96%) [5]. With regard to their review, our work adds with 1133 patients the largest cohort to literature that has been tested with both ID NOW™ and RT-PCR. With a sensitivity of 83.33% our results confirm their findings. However, five of their included studies show a high risk of bias as they do not state patient symptoms or previous testing. During the period of this study, all patients presented to the ED or shock room were routinely tested using ID NOW™ and RT-PCR. Hence, a bias can be excluded. One patient who caused a false-negative ID NOW™ result showed no COVID symptoms at the time of admission. The RT-PCR initially revealed a *C*_T_-value of 37.43, which decreased to 20.9 2 days later. Hence, a transmissibility at the time of admission would be considered as unlikely by today’s standards [14, 15]. Out of the five patients that received a true-positive ID NOW™, two patients had a recent COVID-19 history and one patient suffered from fever and headache. Concluding, the ID NOW™ POCT device is a reliable initial screening method for emergency and shock room patients. However, patients need to be re-evaluated on a regular basis as early infections can be missed.

The main advantage of a POCT device within an emergency department is the generation of COVID-19 test results within minutes. This has not only direct financial benefits but also decreases turnaround times for diagnostics, therapy, and preparation for surgery. While cost evaluation analyses on other respiratory tract infections, such as influenza virus infection, suggest major cost savings by POCT, there is limited evidence for COVID-19 [16–18]. A deterministic decision-analytic model showed cost savings by a COVID-19 antigen test of 210€, mainly based on a reduction of unnecessary bed blocking [8]. We have shown that the initially more expensive ID NOW™ allows staff to save on PPE, OR time, and empty isolation beds. Other studies suggest that, if conventional laboratory testing can be provided within 6 h, the benefits of POCT might be reduced [19]. However, the setting of a traumatological emergency department requires fast diagnosis, decision-making, and in many cases surgery earlier than 6 h. Hence, a POCT device seems to be indispensable. Alternatively, antigen testing can be considered as initial screening method, but it is limited by a low sensitivity detecting mainly patients with a high viral load [20, 21]. Particularly, in an emergency department, which generates patient admissions with hospital stays for numerous days, it seems negligent to choose a test modality with limited reliability. Alternatively, Xpert Xpress (Cepheid Inc.) is a rapid RT-PCR presenting diagnostic results with similar accuracy as ID NOW™ within 30–45 min [21, 22]. The main disadvantage seems to be the prolonged testing time, which makes additional protection and precautionary measures necessary. Other authors report increased test reliability in combination with immunoglobulin G antibody tests or chest computed tomography (CT), which again increases costs and labour resources [23, 24]. Furthermore, a recent meta-analysis determining sensitivity and specificity of chest CT for COVID diagnosis shows with 87% (95% CI 85–90%) and 46% (95% CI 29–63%) inferior results [25]. Recent research also focusses on artificial intelligence driven screening based on quickly available vital signs and routine blood testing achieving only a maximum sensitivity of 84.1% and specificity of 73.3% [26]. Summarizing, rapid molecular POCT device seems to be the best available screening method with a very high specificity and acceptable sensitivity for the use in a traumatological emergency department and shock room with limited time for diagnosis and emergency treatment. The advantage of ID NOW™ is the reduced testing time. However, if used as single screening method, there is a residual risk of false-negative test results during early stages of infection. Hence, clinical suspicion, re-evaluation, and re-testing are inevitable to prevent in-hospital COVID-19 outbreaks.

The study is limited by the dynamic pandemic situation during the study time, with varying incidences and virus variants. Furthermore, workflows are described for a German hospital and might differ significantly outside Germany. Accordingly, costs calculated are based on bulk orders negotiated by the in-hospital purchasing department. Again, costs may vary internationally. Finally, many additional works steps such as post-COVID-19 room cleaning, waste, work time for putting on the PPE and personnel costs in cases of infection and quarantine are not considered within this model. Future calculations of cost-efficiency of diagnostic testing for infectious diseases with epidemic potential should combine direct costs and time to result to calculate the value-based effect expressed in euro-hours as described before for emergency room settings [27].

In the opinion of the authors, the strongest advantage of POCT ID NOW™ lies in the diagnostic speed and high specificity. Particularly, during shock room treatment, a fast exclusion of a COVID-19 infection is of utmost importance as fast therapeutic algorithms are not slowed down by protective procedures. Furthermore, early omission of PPE saves recourses and staff capacities. However, rapid RT-PCR can be considered as alternative diagnostic tool in cases of less urgent patients. For the sake of practicality, we did not implement another POCT within our ER.

In conclusion, our study adds to literature the largest cohort of patients tested with both ID NOW™ and RT-PCR, confirming a sensitivity of 83.33% and a very high specificity of 99.82%. While rapid molecular testing can initially be slightly more expensive than RT-PCR, costs and workload can greatly be reduced particularly in an ED, while workplace safety is increased. The data of this study support the use of rapid molecular POCT device within an ED.

## Data Availability

The datasets used and analysed during the current study are available from the corresponding author on reasonable request.
